# Prohibitin (PHB) inhibits apoptosis in rat granulosa cells (GCs) through the extracellular signal-regulated kinase 1/2 (ERK1/2) and the Bcl family of proteins

**DOI:** 10.1007/s10495-013-0901-z

**Published:** 2013-10-06

**Authors:** Indrajit Chowdhury, Winston E. Thompson, Crystal Welch, Kelwyn Thomas, Roland Matthews

**Affiliations:** 1 Department of Obstetrics and Gynecology, Reproductive Science Research Program, Morehouse School of Medicine, 720 Westview Drive Southwest, Atlanta, GA 30310 USA; 2 Department of Anatomy and Neurobiology, Morehouse School of Medicine, Atlanta, GA 30310 USA

**Keywords:** Prohibitin, Granulosa cells, Survival, Apoptosis, Mitochondria

## Abstract

Mammalian ovarian follicular development is tightly regulated by crosstalk between cell death and survival signals, which include both endocrine and intra-ovarian regulators. Whether the follicle ultimately ovulates or undergoes atresia is dependent on the expression and actions of factors promoting follicular cell proliferation, differentiation or apoptosis. Prohibitin (PHB) is a highly conserved, ubiquitous protein that is abundantly expressed in granulosa cells (GCs) and associated with GC differentiation and apoptosis. The current study was designed to characterize the regulation of anti-apoptotic and pro-apoptotic factors in undifferentiated rat GCs (gonadotropin independent phase) governed by PHB. Microarray technology was initially employed to identify potential apoptosis-related genes, whose expression levels within GCs were altered by either staurosporine (STS) alone or STS in presence of ectopically over-expressed PHB. Next, immunoblot studies were performed to examine the expression patterns of selective Bcl-2 family members identified by the microarray analysis, which are commonly regulated in the intrinsic-apoptotic pathway. These studies were designed to measure protein levels of Bcl2 family in relation to expression of the acidic isoform (phosphorylated) PHB and the components of MEK-Erk1/2 pathway. These studies indicated that over-expression of PHB in undifferentiated GCs inhibit apoptosis which concomitantly results in an increased level of the anti-apoptotic proteins Bcl2 and Bclxl, reduced release of cytochrome c from mitochondria and inhibition of caspase-3 activity. In contrast, silencing of PHB expression resulted in change of mitochondrial morphology from the regular reticular network to a fragmented form, which enhanced sensitization of these GCs to the induction of apoptosis. Collectively, these studies have provided new insights on the PHB-mediated anti-apoptotic mechanism, which occurs in undifferentiated GCs through a PHB → Mek-Erk1/2 → Bcl/Bcl-xL pathway and may have important clinical implications.

## Introduction

Apoptosis is a genetically controlled cellular suicide mechanism that plays a crucial role in the development and defense of homeostasis in each organ system. In the ovary, more than 99 % of follicles disappear, primarily due to apoptosis of granulosa cells (GCs) during follicular growth and development [1, 2]. Ovarian GCs play an important physiological role in supporting the development and selection of the ovarian follicle by controlling oocyte maturation and by producing the steroid hormones, estradiol and progesterone, that are critical for maintenance of the ovarian cycle. Both biochemical and morphological characteristics of apoptosis have been observed in the GCs of atretic follicles [3–5]. However, the mechanism by which GCs escape apoptosis during gonadotrophin independent phase is poorly understood. For these reasons, analyses of the molecular events occurring during gonadotrophin independent phase development of GC are pivotal to our understanding of how these cells contribute to the modulation of processes critical for oocyte development. To date, many apoptosis-related factors have been implicated in follicular atresia, including death ligands and receptors, intracellular pro- and anti-apoptotic molecules, cytokines and growth factors that regulate functionally distinct phases (initiation, effector and degradation) of apoptosis. During the initiation phase of apoptosis, cells receive death inducing signals from MEK to ERK pathway which are executed by degradation of specific target proteins and inter-nucleosomal/chromosomal DNA [6, 7]. Currently, our knowledge of transcriptional and translational cascades involved in survival signaling during folliculogenesis remains incomplete. Studies from our laboratory have shown that prohibitin (PHB) is one of the survival factors in undifferentiated rat GCs [8–10].

Prohibitins [PHB (prohibitin, b cell associated protein 32) and repressor of estrogen receptor activity (REA, prohibitin PHB2, b-cell associated protein 37)] are highly conserved protein families that are thought to play specific roles in cell cycle control, differentiation, senescence and antiproliferative activity [10]. A growing body of evidence has implicated a role for PHB in mitochondrial structure, function and inheritance [8–14]. Previously published experimental observations [8, 9] suggested that PHB may be a cell survival or anti-apoptotic factor that is likely to play an important role in cell fate decision and in mitochondrial integrity/cellular homeostasis. However, details about the anti-apoptotic mechanisms involving PHB at the transcriptional and translational level in undifferentiated GCs are still unclear.

In order to elucidate the anti-apoptotic mechanisms mediated by PHB in GCs isolated from immature rat follicles, the staurosporine (STS) induced apoptotic model was analyzed using Affymetrix microarray technology. This experimental strategy was utilized to identify potential apoptosis-related genes, whose expression levels within the undifferentiated GCs were altered by the PKC inhibitor, STS alone or in presence of over-expressed PHB. STS at higher (micro molar) concentration has been extensively used in vitro as an initiator of apoptosis in many different cell types [15, 16] including GCs in our previously published studies [8], whereas at lower (nano molar) concentration it is an inhibitor of PKC [15, 16]. Based on these studies, induction of apoptosis in undifferentiated GCs was achieved using 1 μM STS within the period of 4 h [8]. Using this model of STS induced apoptosis in undifferentiated GCs microarray studies identified several members of the Bcl2 family. Immunoblot studies were then performed to analyze the expression levels of selective Bcl-2 family members, which are commonly regulated in the intrinsic-apoptotic pathway at the protein level. Parallel studies were performed to examine expression of the acidic (phosphorylated) isoform of PHB and its relationship with the MEK-Erk1/2 pathway. In addition, we also analyzed the role of PHB in relation to STS induced mitochondrial morphological changes.

## Materials and methods

### Granulosa cell (GC) cultures

Primary undifferentiated GCs were isolated from immature (23 days old) Sprague–Dawley rat ovaries as previously described [8]. Ovaries were collected into serum-free medium (4F), which consists of 15 mM HEPES, pH 7.4, Dulbecco’s modified Eagles’s medium/F-12 with transferrin (5 μg/ml), human insulin (2 μg/ml), hydrocortisone (40 ng/ml), and antibiotics. After incubating the ovaries at 37 °C in 4F medium containing 0.5 M sucrose and 10 mM EGTA for 30 min, the ovaries were washed in fresh 4F medium. Undifferentiated GCs were collected from the ovaries by puncturing follicles with a 25-gauge hypodermic needle, and cells were dispensed into 4F medium supplemented with 10 % FBS (GIBCO BRL, Grand Island, NY, USA), and incubated in a humidified atmosphere of 5 % CO_2_ at 37 °C. The GCs isolated from sexually immature 23–25-day-old rats are referred to as undifferentiated because they lack the presence of functional LH receptor and do not produce estrogen or progesterone under basal conditions. However, these cells respond to FSH with respect to the production of cAMP and induction of LH receptor activation of the estrogen and progesterone biosynthetic pathways [17]. All experimental protocols were submitted to the Institutional Animal Care and Use committee and were in accordance with the guidelines of the National Institutes of Health and the U.S. Department of Agriculture. This committee approved all animal care handling procedures described in the present study.

### Generation of recombinant adenoviral plasmid vectors

The PHB gene was amplified by PCR using rat ovarian cDNA template as described previously by Chowdhury et al. [8, 9]. The plasmid in the bacteria was amplified and purified using a plasmid maxiprep system (Qiagen, Valencia, CA, USA). The complete adenovector was linearized and used for transfection of Ad293 cells (human embryonic kidney cell line), where viral particles were further amplified, purified, and titered according to the manufacturer instruction [8, 9, 18]. The empty adenovirus (Ad) vector was used as a control.

The shRNA cassettes that specifically targeted specific motifs in the PHB sequence were designed through shRNA Target Finder (GenScript, Scotch Plains, NJ, USA) as described previously by Chowdhury et al. [8, 9].

### Adenoviral (Ad) infection of granulosa cells (GCs)

The undifferentiated GCs were grown on 6-well culture dish (~2 × 10^6^ cells/well) in 4F media supplemented with 10 % FBS. Subsequently, medium was removed and cells were washed twice with 4F (antibiotics-free) and infected with or without adenoviral vectors (Ad-eGFP: adenovirus with GFP or Ad-eGFP-PHB: adenovirus GFP with sense cDNA PHB; Ad-scrambled: adenovirus with scrambled sequence RNA; Ad-eGFP-shPHB: adenovirus with small interfering RNA designed for knockdown of PHB) at a multiplicity of infection (MOI) of 10 plaque-forming units per cell (pfu/cell) (based on our previous studies 8, 9) with occasional rocking. After 2 h of incubation, media was replaced with fresh 4F media without FBS and incubated for 24 h. Infected GCs showed 95–100 % GFP expressions at 10 pfu/cell (data not shown). Twenty-four hours after exposure to adenoviruses, the media was replaced with fresh 4F media without FBS in the presence or absence of MEK inhibitor (PD98059; 20 μM) for 1 h. The cells were then treated with or without STS (1 μM) for 2 h. Total RNA and proteins were isolated for the further analysis. All the doses and time for viral infection were based on our previous experiments [8, 9].

### Induction of apoptosis

The induction of GCs apoptosis were done in serum-free medium in the presence of STS at a dose of 1 μM concentration for 2 h. STS concentration and time response were used based on our previous experiments [8]. Following STS treatment the percentage of apoptosis was determined by nuclear staining with Hoechst 33248 stain (12.5 ng/ml; Sigma) as described by Chowdhury et al. [8, 9]. At least 250–300 cells were counted for each data point.

### Caspase enzyme activity

Caspase-3 activity was measured using a colorimetric assay kit (CaspACE-colorimetric; Promega, Madison, WI, USA) as described previously by Chowdhury et al. [8, 9]. Caspase-3 activity was calculated in picomoles per hour per microgram of protein and plotted as percentage of control.

### Microarray sample preparation and hybridization

To analyze the differentially expressed mRNA profiles at the end of each experiments, cells were washed with ice cold PBS and stored at −70 °C until RNA was prepared. Total RNA was extracted with TRIzol Reagent (Life Technologies, Rockville, MD, USA), purified (RNAqueous Kit, Ambion, Austin, TX, USA) and converted to double-stranded cDNA (Invitrogen, Superscript Choice System, Carlsbad CA, USA) using a T7-(dT)24 primer. The double-stranded cDNA was isolated using Phase Lock Gels (Eppendorf, Westbury, NY, USA)–phenol/chloroform/isoamyl alcohol (Sigma, St. Louis, MO, USA). cRNA was synthesized using a RNA transcript labeling kit (Enzo Diagnostics, Farmingdale, NY, USA). Biotin-labeled cRNA was purified using a GeneChip Sample Cleanup Module (Affymetrix Inc, Santa Clara, CA, USA) and then quantified using a spectrophotometer. Next, twenty micrograms (20 μg) of the in vitro transcription product was fragmented by placing at 94 °C for 35 min in fragmentation Buffer. Following fragmentation, 15 μg of the biotinylated cRNA was hybridized to an Affymetrix Rat Genome U34A GeneChip with 8799 probe sets (genes). The chips were hybridized at 45 °C for 16 h, and then washed, stained with streptavidin–phycoerythrin, and scanned according to guidelines provided by the manufacturer.

### Microarray data processing

Data analysis was performed by Affymetrix Microarray Suite (MAS) 5.0 software. The microarray suite references the experimental file to select an analysis algorithm for a cell intensity file that generates a gene chip file. Single array analysis was used to build the databases of gene expression profiles. Affymetrix GCOS software was used to normalize and analyze the data. Detection *P* value (set at *P* < 0.05) was used to statistically determine whether a transcript is expressed on the chip. The software generated a present (P), marginal (M), or absent (A) call for each transcript based on the *P* value. To obtain differentially expressed genes for each condition, Affymetrix GeneChip Operating Software (GCOS) was used to compare each of the STS treated alone or in presence of Ad-eGFP or Ad-eGFP-PHB arrays to that of the control arrays. Absolute calls (P, M and A) and the average difference (RNA abundance) for each gene were then imported into Genespring software (Silicon Genetics, Redwood City, CA, USA) for a self-organizing map (SOM) cluster analysis by dividing the genes of control versus experimental clusters based on the expression patterns. By combining the fold change and the present calls derived from the comparisons, we obtained a list of differentially expressed genes for each condition. Differential expression was calculated as the increase between the controls and STS treated groups (i.e. control versus STS treated group, Ad-eGFP or Ad-eGFP-PHB alone versus Ad-eGFP or Ad-eGFP-PHB with STS). A gene was considered differentially expressed when the standard deviation of the signal increase or decrease was significantly smaller than the absolute change in average difference and the calculated confidence level of a gene was set greater than 95 % (*P* < 0.05 based on unpaired *t* test). The general view of the effect of the PHB on gene expressions in the GCs were obtained by SOM cluster analysis using Genespring software (Silicon Genetics) on replicate samples. Selected clusters were examined for biological function and pathway analysis using Affymetrix Netfix Analysis Center (http://www.affymetrix.com). Netfix detailed and annotated individual probe sets based on biological and molecular function or cellular localization using the Gene Ontology public database.

### Assessment of mitochondrial changes

For the assessment of mitochondrial integrity, GCs were stained with 200 nm MitoTracker Red solution in 4F medium at 37 °C temperatures for 15 min as described previously by Chowdhury et al. [8, 9], and were analyzed using a laser scanning confocal microscope imaging system (Olympus Corp., Melville, NY, USA).

### Isolation of S-100 fraction and mitochondria

S-100 (cytosolic) fractions and mitochondria were prepared as described by Chowdhury et al. [8, 9]. Protein expression levels in the respective cellular fractions were analyzed by Western blot.

### Western blot analysis

GC protein extracts obtained from different treatment conditions were subjected to one- or two-dimensional gel electrophoresis. The procedures used for one- and two-dimensional gel electrophoresis, protein transfer, and blotting have been described previously [8, 9, 13]. For one-dimensional gel electrophoresis, equal amounts of protein (25 μg) were applied to each lane. For two-dimensional gel electrophoresis, eighty micrograms of protein purified from mitochondrial fractions isolated from cultured GCs after treatment were focused in the first dimension on IPG pH gradient 4–7 strips for 60 kV-h using a Bio-Rad Protean IEF Cell and second dimension followed by the Western blotting procedure and PHB antibody to detect protein spots corresponding to PHB. Primary antibodies used were rabbit polyclonal PHB (1:1,000; Neomarks, Fremont, CA, USA), mouse monoclonal cleaved caspase 3 (1:1,000; Cell Signaling, Beverly, MA, USA), mouse monoclonal cytochrome c (1:1,000; Cell Signaling, Beverly, MA, USA), rabbit polyclonal Bcl2 (1:1000; Cell Signaling, Beverly, MA, USA), rabbit polyclonal Bclxl (1:1,000; Cell Signaling, Beverly, MA, USA), rabbit polyclonal Bax (1:1,000; Cell Signaling, Beverly, MA, USA), rabbit polyclonal Bak (1:1,000; Cell Signaling, Beverly, MA, USA), rabbit polyclonal total Erk1/2 and pErk1/2 (1:1,000; Cell Signaling, Beverly, MA, USA), rabbit polyclonal porin (1:1,000; Cell Signaling, Beverly, MA, USA) and cyclophilin-a (1:1,000; Neomarks, Fremont, CA, USA). Membranes were incubated with the appropriate secondary antibody for 2 h at room temperature, and antibody binding was detected by chemiluminescence (Pierce, Rockford, IL, USA). Results of representative chemiluminescence were scanned and densitometrically analyzed using a Power Machintosh Computer (G3; Apple Computer Inc., Cupertino, CA, USA) equipped with a ScanJet 6100C Scanner (Hewlett-Packard Co., Greeley, CO, USA). Quantification of the scanned images was performed according to the NIH Image version-1.61 software (National Institute of Health, Bethesda, MD, USA).

### Quantification of the pro-apoptotic Bcl2 protein versus anti-apoptotic Bcl2 protein ratio

Quantitative analysis of the pro-apoptotic Bcl2 protein versus anti-apoptotic Bcl2 protein expression were performed using a scanning densitometer and Multianalyst Software Version 1.0.2 (Biorad, Munich, Germany) as described by Prokop et al. [19]. Standardization of protein loading was achieved as follows: (1) protein measurements of all samples were performed using the Bio-Rad Protein assay kit and equal amounts of protein (25 μg per lane) were loaded on the gel; (2) transfer efficiency of the Western blots was routinely checked by staining the membranes with 0.5 % Ponceau Red in 1 % acetic acid; (3) for chemiluminescent detection, films were exposed for exactly the same length of time, and were optimized for each antibody used in this study; and (4) detection of Bax, Bak, Bclxl and Bcl2 were performed separately using the same membrane. This procedure facilitated quantitative determination of the protein ratios in the same sample (on the same lane of the gel). Values of protein expression are given in arbitrary units in percentages after normalization to cyclophilin A. Linearity of protein detection was checked for Bcl2 family protein using standard cell extracts as provided by the Cell Signaling, Beverly, MA.

### Statistical analysis

All experiments were replicated a minimum of three times, unless otherwise stated. Data are expressed as mean ± SEM of three experiments. Statistical analysis was performed by one-way ANOVA using SPSS version 11.0 software (SPSS, Chicago, IL, USA). Multiple comparisons were done by Newman–Keuls’ test. Differences were considered significant at *P* ≤ 0.05.

## Results

### Over expression of PHB inhibits apoptosis in undifferentiated GCs

**Fig. 1:**
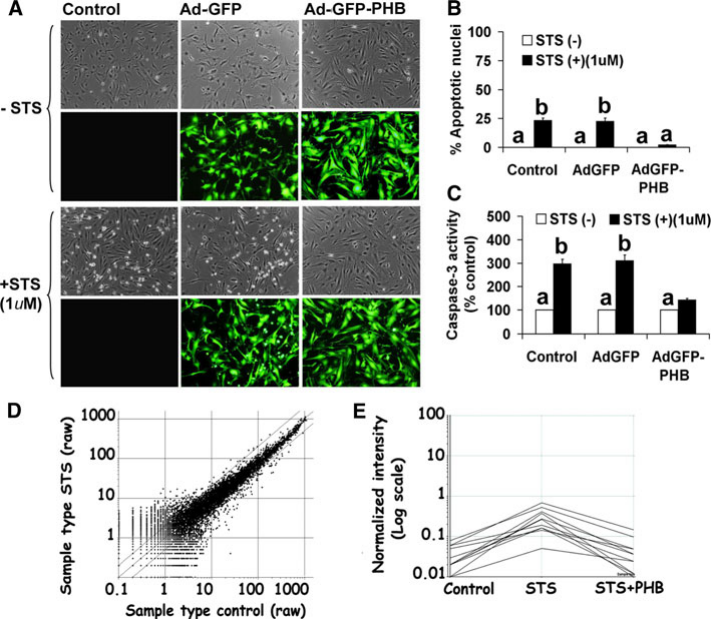
Effects of recombinant adenovirus-directed overexpression of PHB on PKC inhibitor, STS induced apoptosis in undifferentiated rat GCs. Undifferentiated GCs were infected with sense adenovirus-eGFP-PHB (MOI = 10) or an adenovirus-eGFP vector control (MOI = 10) for 2 h and maintained in culture for 24 h along with uninfected parallel control groups. Thereafter, GCs were treated with STS (1 μM) for 2 h. **A** Live cell photographs were taken under an inverted epifluorescence microscope at ×400 magnification showing green fluorescence for the overexpressed eGFP-PHB or eGFP alone along with phase contrast pictures at 2 h in presence or absence of STS. **B** Data shown represent the percentage of cells displaying nuclear morphologic changes characteristic of apoptosis. **C** Data representing the caspase 3 activity as % of control groups in cytosolic protein extracts from GCs after completion of treatments measured using the spectrophotometric substrate DEVD-pNA(D). **D** cDNA Microarray analysis profile of total mRNA of STS induced changes in presence or absence of recombinant adenovirus-directed over-expressed PHB in undifferentiated GCs. Data is represented by as a *scatter plot* of the expression of all the genes (8799) present on Affymetrix Rat Genome (RGU34A) Gene Chip showing expression twofold or more following STS treatment in presence or absence of PHB. *Fold change lines* represent genes that increase or decrease in signal intensity by twofold or more. **E** cDNA Microarray analysis of apoptotic genes expression that increased significantly with STS treatment and decreased significantly with in presence of PHB (see Table 1). Significance based on twofold change increase or decrease. All numerical values are represented as mean ± SEM of three individual experiments (n = 3). The values show overall significance in one way ANOVA test (*P* < 0.001). All groups are significantly different (*P* < 0.05, Newman–Keuls’ test) except the groups indicated by *a* and *b*

As shown in Fig. 1A, apoptotic cell death with STS was potent and significant (*P* < 0.05; Newman–Keuls’ test) in rat primary GC culture system. STS treatment resulted in cell detachment, loss of cell processes, membrane shrinkage, as evidenced by curling of cells and formation of apoptotic bodies. Staining (Hoechst 33248) of nucleus showed significant (23–30 %, *P* < 0.05; Newman–Keuls’ test) nuclear morphologic changes with chromatin condensation and fragmentation into apoptotic bodies after STS treatment with or without Ad-eGFP infection of the GCs (Fig. 1B). In marked contrast, less than 5 % Ad-eGFP-PHB-infected cells were apoptotic compared to their parallel control groups. Based on these results, we used 1 μM dose of STS treatment of GCs for 2 h for all other experimental studies.

Caspase-3 has been shown to be a potent effector caspase that targets several specific cellular proteins causing a number of changes associated with cell death. Therefore, the apoptotic nature of cell death was further verified by examining the biochemical differences observed in the Ad-eGFP or Ad-eGFP-PHB infected cells in presence or absence of STS by quantification of caspase-3-enzyme activity. Analysis of cytosolic caspase-3 enzymatic activities after STS treatment for 2 h showed a significant (*P* ≤ 0.05) increase (three to fourfold) in caspase-3 activity in GCs infected with empty Ad-vector (Ad-eGFP) compared to Ad-eGFP-PHB infected group (Fig. 1C). In agreement with the morphological differences observed and quantitative measures of the extent of apoptosis in the undifferentiated GCs, these studies suggest that exogenous over-expression of PHB provides marked inhibition against STS-induced apoptosis.

### Over expression of PHB increases the levels of expression of anti-apoptotic Bcl2 family members in undifferentiated GCs

To explore the mechanism through which PHB is able to prevent apoptosis in undifferentiated GCs treated with STS to activate the intrinsic apoptotic pathway, the microarray experiments were used to generate a unbiased global view of gene expression patterns in the respective experimental samples (Fig. 1D). To assess inter-experimental differences in intensity of gene expressions, replicate experiments were compared for each experimental group. To elucidate the magnitude of the changes in gene expression and the functional significance of PHB in STS-induced gene regulation in the undifferentiated GCs, we subjected the genes from the three SOM clusters (each control to the STS samples and STS to STS + PHB samples) to pathway analysis using the Affymetrix web based program NetAffx [20]. Using NetAffx, we examined genes with annotations related to biological processes. The genes regulated during STS and STS with PHB treatment fell into several broad categories including development, morphological, cell growth and maintenance, signal transduction, metabolism, transport and apoptosis. Treatment of the GCs with Ad-eGFP-PHB resulted in a reduction in the expression levels of many of the apoptotic inducing genes by 50 % or more. Therefore, we restricted our analysis to apoptotic related gene products (Fig. 1E). Based on the apoptotic gene cluster analysis, genes were selected that showed ~1.5 to twofold difference on each individual probe set. The selected gene names and the GenBank accession numbers are included in Table 1 and their expression profiles under the experimental conditions are shown in Fig. 1E. A large proportion of these apoptotic genes appeared to be associated with the Bcl family. Interestingly, a number of major pro-apoptotic regulators including Bax and Bak were also specifically regulated upon STS treatment with almost identical expression profiles. After initial induction with Ad-eGFP-PHB for 24 h, both Bax and Bak are down-regulated. Furthermore, caspase-3 gene expression that was specifically activated upon STS treatment was down-regulated in the Ad-eGFP-PHB treated groups. Compared to pro-apoptotic regulators, two major anti-apoptotic gene transcripts Bcl2 and Bclxl were up-regulated in GCs infected with Ad-eGFP-PHB and treated with or without STS.

**Table 1:** Microarray analysis of gene expression patterns in rat undifferentiated granulosa cells stimulated by PKC inhibitor, STS in presence and absence of adenovirus directed over-expressed prohibitin-1 (Ad-eGFP-PHB1)

Accession number	Name	STS	STS + Ad-eGFP-PHB1
NM_031851.2	Prohibitin-1	2.33	4.93
AF155236.1	ERK1b	1.38	2.11
NM_012840.1	Cyt C	19.63	9.32
NM_012922.2	Caspase-3	2.62	1.35
NM_016993.1	Bcl2	1.05	2.24
NM_001033670	Bcl_XL_	1.35	2.35
NM_017059.1	Bax	1.57	0.57
AF259504	Bak	1.48	0.74

**Fig. 2:**
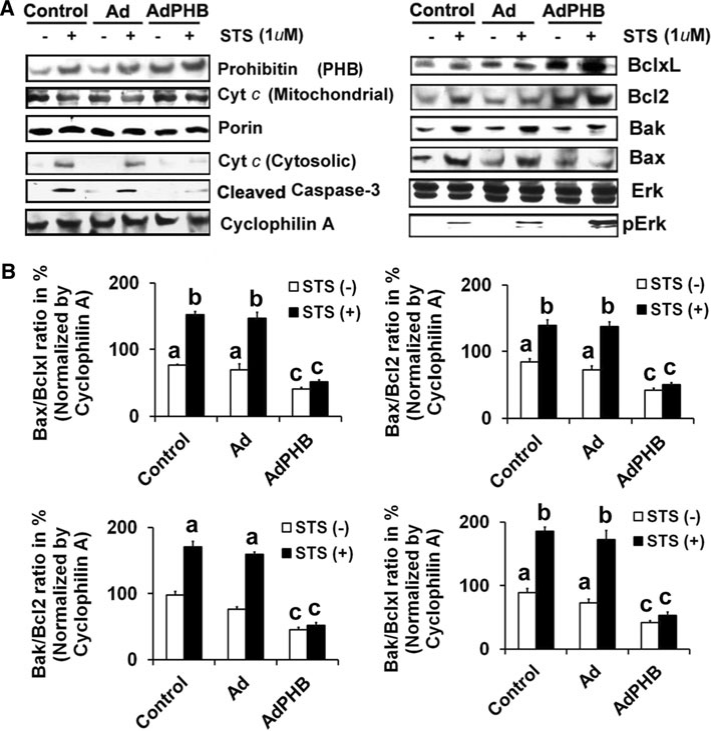
Western blot analysis of recombinant adenovirus-directed over-expression of PHB on PKC inhibitor, STS induced apoptosis related change in protein profile in undifferentiated rat GCs. Undifferentiated GCs were infected with sense adenovirus-eGFP-PHB (MOI = 10) or an adenovirus-eGFP vector control (MOI = 10) for 2 h and maintained in culture for 24 h along with uninfected parallel control groups. Thereafter, GCs were treated with STS (1 μM) for 2 h. **A** Representative Western blots of protein levels in GCs treated by STS in presence and absence of adenovirus directed over-expressed PHB for PHB, cleaved caspase 3, cytochrome c (mitochondrial and cytosolic), Bcl2, Bclxl, Bax, Bak, total Erk1/2 and pErk1/2. Porin and cyclophilin A were used as internal controls for mitochondria and cytosol, respectively. Data is representative of three individual experiments (n = 3) that were performed for each individual group. **B** The *bar graphs* representing the % mean ± SEM of Bax/Bclxl, Bax/Bcl2, Bak/Bclxl and Bak/Bcl2 ratios of protein levels normalized by cyclophilin A. Data are representative of three individual experiments (n = 3) that were performed for each individual group. The values show overall significance as indicated in one way ANOVA test (*P* < 0.001). All groups were significantly different (*P* < 0.05, Newman–Keuls’ test) except the groups indicated by *a*–*c*

Based on the gene array analyses, we selected eight candidate genes and tested their influence on cell death versus cell survival mechanisms. First, we explored whether over-expression of PHB prevented apoptosis at the mitochondrial level by inhibiting mitochondrial cytochrome c release to cytosol and concomitant activation of down-stream caspase-3, as part of the anti-apoptotic mechanism. As shown in Fig. 2A, a majority of cytochrome c was released from mitochondria to the cytosol in control/or Ad-eGFP infected GCs treated with STS. In contrast, only a small amount of cytochrome c was released from mitochondria to cytosol in Ad-eGFP-PHB infected cells treated with STS for 2 h. Similar to the differential release of mitochondrial cytochrome c to cytosol in different treated groups, the activation of caspases-3 also showed a differential pattern with positive relationship to cytosolic cytochrome c release. STS treatment of control/or Ad-eGFP infected cells revealed more than three to fourfold higher activation of caspases-3 expression compared to untreated controls or Ad-eGFP-PHB infected GCs. Thus, over-expression of PHB was able to block downstream apoptotic events through inhibiting the release of cytochrome c. Next, we examined the relative expression levels of pro- and anti-apoptotic Bcl family proteins including Bcl2, Bclxl, Bax and Bak. The expression of Bcl2, Bcl-xl, Bax and Bak showed significant variations in control and Ad-eGFP/Ad-eGFP-PHB infected groups with or without STS treatment (Fig. 2A). Interestingly, an enhanced level of Bcl2 and Bclxl protein expression was revealed in Ad-eGFP-PHB infected cells. Moreover, in the Ad-eGFP-PHB infected cells, Bax and Bak expression were suppressed even in presence of STS. The quantitative expression analysis represented by Bax/Bcl2, Bax/Bclxl, Bak/Bcl2 and Bak/Bclxl ratios are shown in Fig. 2B. In confirmation of our initial observations, we found that Bax/Bcl2, Bax/Bclxl, Bak/Bcl2 and Bak/Bclxl ratios were significantly two to threefolds lower in Ad-eGFP-PHB infected GCs. In addition, we also analyzed the expression levels of total and phosphorylated Erk, since the Erk pathway genes were also found to be regulated by gene-array analysis. In Ad-eGFP-PHB infected cells, we also observed enhanced expression of pErk compared to parallel control or Ad-eGFP infected cells or treated with STS (Fig. 2A). These results suggest that over-expression of PHB may, at least in part, exert its protective effects by suppressing STS-induced apoptotic gene expression (Bax and Bak) and enhancing expressions of the anti-apoptotic proteins (pErk, Bcl2 and Bclxl) in GCs.

### Over expression of PHB activates expression of anti-apoptotic factors through phosphorylated Erk (pERK) in undifferentiated GCs

**Fig. 3:**
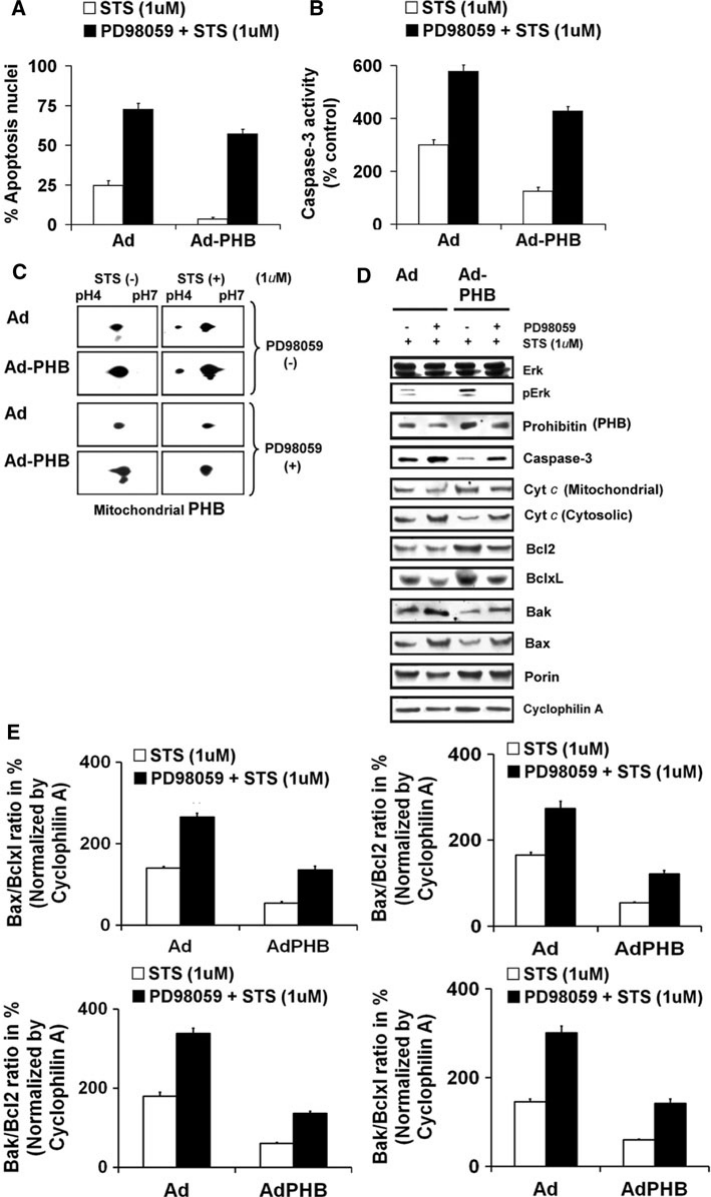
Western blot analysis of recombinant adenovirus-directed over-expression of PHB in presence or absence of MEK inhibitor (PD98059) on the PKC inhibitor STS induced apoptosis in undifferentiated rat GCs. Undifferentiated GCs were infected with sense adenovirus-eGFP-PHB (MOI = 10) or an adenovirus-eGFP vector control (MOI = 10) for 2 h and maintained in culture for 24 h followed by treated with MEK inhibitor (PD98059) for 1 h. Thereafter, undifferentiated GCs were treated with STS (1 μM) for 2 h in serum free media. **a** Data shown represent the percentage of cells displaying morphological alteration of apoptosis based on quantification of nuclear morphologic changes. **b** Graphically, data represent the caspase 3 activity as % of control groups in cytosolic protein extracts prepared from GCs after completion of treatments and measured using the spectrophotometric substrate DEVD-pNA(D). **c** 2D-Western blot analyses of mitochondrial protein levels for PHB in undifferentiated GCs after various treatments as indicated. **d** Representative Western blots of protein expression levels for PHB, cleaved caspase 3, cytochrome c (mitochondrial and cytosolic), Bcl2, Bclxl, Bax, Bak, total Erk1/2 and phosphor ERK1/2 (pErk1/2) for the various treatment groups. Porin and cyclophilin A were used as an internal control for mitochondria and cytosol, respectively. **e** The *bar graphs* shown represent the % mean ± SEM of Bax/Bclxl, Bax/Bcl2, Bak/Bclxl and Bak/Bcl2 ratios of protein levels normalized to cyclophilin A from three replicate experiments. Data shown are a representative of three individual experiments (n = 3) were performed for each individual group. All numerical values are represented as mean ± SEM of three individual experiments (n = 3). The values shown indicate an overall significance as determined by the one way ANOVA test (*P* < 0.001). All groups are significantly different (*P* < 0.05, Newman–Keuls’ test)

We explored whether over-expression of PHB inhibited apoptosis through activation of the MEK-Erk-signaling pathway. As shown in Fig. 3A, B, apoptotic cell death and caspase-3 activity were most potent and significant (*P* < 0.05; Newman–Keuls’ test) in presence of the MEK-inhibitor (PD98059) in STS treated GCs. The MEK-inhibitor in presence of STS also significantly (*P* < 0.05; Newman–Keuls’ test) promoted cell death and enhanced caspases-3 activity in over-expressed PHB GCs when compared to the parallel control GCs without MEK inhibitor (Fig. 3A, B). Interestingly, in GCs, pretreatment of MEK-inhibitor (PD98059) at 20 μM for an hour did not elicit any cell death (Table 2). Our unpublished results also shown that GCs were treated with PD98059 for 4 h did not increase in cell death [21].

**Table 2:** Effects of pretreatment of MEK inhibitor, PD98059 in rat undifferentiated granulosa cells

Group	Time (h)	Apoptosis (%)	Caspase-3 activity (% control)
Control	0	0 ± 0	100 ± 0
PD98059	0	0 ± 0	100 ± 0
Control	1	0 ± 0	100 ± 0
PD98059	1	0 ± 0	100 ± 0
Control	2	0 ± 0	100 ± 0
PD98059	2	2 ± 1.15	103 ± 1.53
Control	3	0 ± 0	100 ± 0
PD98059	3	2.33 ± 1.2	103.67 ± 1.2

We further analyzed whether mitochondrial PHB coordinates the signaling between MEK1 and ERK1/2 by affecting MEK1 activation in response to STS treatment in primary GCs and whether it affects Bcl family members. As shown in Fig. 3C, 2-D Western blot analysis, revealed that mitochondrial PHB is phosphorylated [8, 10] when the undifferentiated GCs were treated with STS, whereas the phosphorylated form of mitochondrial PHB was inhibited by the MEK inhibitor (PD98059). Next, we analyzed the relative expression levels of pro- and anti-apoptotic Bcl family proteins (Bcl-2, Bclxl, Bax and Bak) under these experimental conditions. The expression levels of Bcl2, Bclxl, Bax and Bak showed significant variations in Ad-eGFP infected cells when compared to Ad-eGFP-PHB infected groups with or without STS treatment in presence or absence of MEK-inhibitor (Fig. 3D). In presence of MEK inhibitor, Ad-eGFP-PHB infected GCs treated with STS showed a marked decreased in Bcl-2 and Bcl-X_L_ protein content and enhanced levels of expressions of Bax and Bak. The quantitative analysis of Bax/Bcl-2, Bax/Bcl-X_L_, Bak/Bcl2 and Bak/Bclxl ratios revealed significant (*P* < 0.05; Newman–Keuls’ test) two to threefold lower protein ratios in Ad-eGFP-PHB infected GCs in presence of MEK inhibitor and STS treatment. These observations correlated with our data on cytochrome c released from mitochondria to the cytosol in the STS treated GCs in presence or absence of MEK inhibitor. In presence of MEK inhibitor, the STS treated undifferentiated GCs are more sensitized even in Ad-eGFP-PHB treated undifferentiated GCs and released high amounts of cytochrome c from mitochondria to cytosol. Moreover, in presence of MEK inhibitor, the activation of caspases-3 is significantly (*P* < 0.05; Newman–Keuls’ test) higher in STS treated undifferentiated GCs, and this caspase-3 activity is directly correlated with the release of cytosolic cytochrome c in agreement with our previous studies [8, 9].

### Knockdown of PHB induces mitochondrial fragmentation and sensitizes undifferentiated GCs to apoptosis

**Fig. 4:**
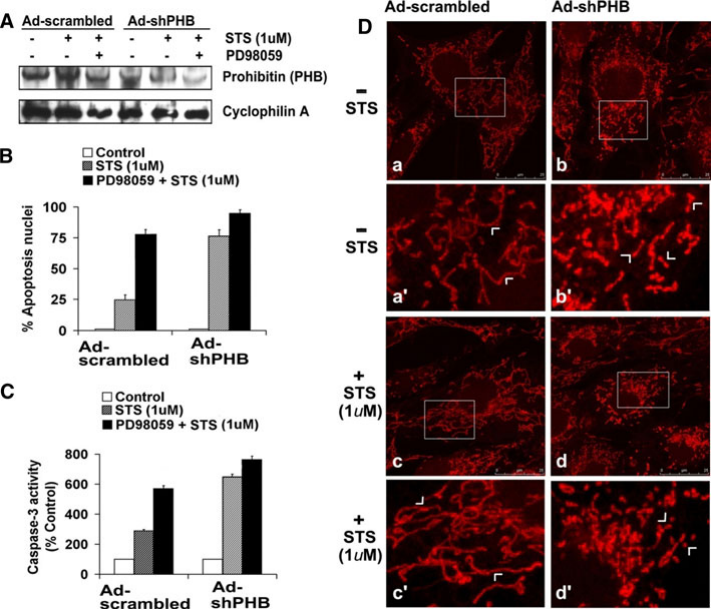
Western blot and morphological analysis of recombinant adenovirus-directed silencing the PHB in presence or absence of MEK inhibitor (PD98059) on PKC inhibitor, STS induced apoptosis in undifferentiated rat GCs. Undifferentiated GCs were transiently infected with AdshPHB (MOI 10) or Ad-scrambled (MOI 10) for 2 h and maintained in culture for 48 h in serum free media followed by treatment with or without MEK inhibitor (PD98059). Thereafter, the GCs were treated with STS (1 μM) for 2 h in serum free media. **A** Representative Western blots of protein expression levels for PHB and cyclophilin A (as an internal control). **B** Data shown represent the percentage of cells displaying morphological alteration of apoptosis based on quantification of nuclear morphologic changes. **C** Data shown represent the caspase 3 activity as % of control groups in cytosolic protein extracts prepared from GCs after completion of treatments and measured using the spectrophotometric substrate DEVD-pNA(D). **D** Representative mitochondrial morphology observed in STS treated or untreated GCs in presence of Ad-scrambled or AdshPHB. White arrows indicate fragmented mitochondria. Representative microscopic analysis from three individual experiments (n = 3) that were performed for each individual group. All numerical values are represented as mean ± SEM of three individual experiments (n = 3). The values show are overall significance determined by one way ANOVA test (*P* < 0.001). All groups are significantly different (*P* < 0.05, Newman–Keuls’ test)

To study the effects of PHB on mitochondrial morphology and its protective role against apoptosis, we knocked down PHB gene expression in undifferentiated GCs using adenoviral shRNA (AdshRNA) (Fig. 4A). Infection with empty Ad-vector alone did not show any changes in PHB levels. As shown in Fig. 4B, shRNA-mediated (AdshRNA) knock-down of PHB sensitized the GCs to STS treatment. In presence of MEK inhibitor (PD98059), the GCs were more sensitized and showed enhanced apoptosis compared to AdshRNA scrambled infected cells (*P* < 0.05; Newman–Keuls’ test). Knock-down of PHB in presence of MEK inhibitor in GCs treated with STS showed increased caspases-3 activity of two to three fold compared to AdshRNA virus infected cells (*P* < 0.05; Newman–Keuls’ test) (Fig. 4C). These results, in part, suggest that MEK is likely to be an important factor regulating apoptosis in these undifferentiated GCs.

To further analyze the effects of PHB on mitochondrial morphology in STS treated GCs, we studied the consequences of knockdown of PHB in presence or absence of STS on mitochondrial morphology by immunofluorescence microscopy. As shown in Fig. 4D, AdshRNA infected undifferentiated GCs have elongated and branch network of mitochondria compared to AdshPHB infected GCs. Interestingly, when AdshPHB infected undifferentiated GCs were treated with STS, the mitochondrial reticulate network were more sensitized and fragmented and appeared as a punctuated form compared to STS treated AdshRNA infected GCs.

## Discussion

This current study demonstrates that PHB plays an anti-apoptotic role in the mitochondrial intrinsic apoptotic pathway and regulates expression of the Bcl family proteins in STS induced apoptotic model of rat ovarian undifferentiated GCs. Moreover, adenoviral directed overexpression of PHB abrogated STS induced cytochrome c release and subsequent activation of caspase 3, thereby preserving the viability of these GCs by tilting the balance in favor of survival. In contrast, shRNA mediated knock-down of PHB using recombinant adenoviral vectors appear to tilt the balance in favor of cell death by affecting the integrity of mitochondrial architecture. Our results further demonstrated that in the mitochondria, phosphorylated PHB prevents expression of two major proapototic factors Bax and Bak through the MEK-Erk-Bcl2/Bclxl pathway in STS induced apoptosis in the undifferentiated GCs. Under these experimental conditions increased expression of anti-apoptotic factor Bcl2 and Bclxl were able to maintain the integrity of the mitochondria architecture. The preservation of cell viability in response to PHB over-expressions is associated with enhanced translocation of PHB into the mitochondria in response to STS induced apoptotic signals [8, 9].

Apoptosis occurs through two major signaling pathways: extrinsic or intrinsic. The intrinsic apoptotic signaling pathway which involves mitochondria results in the release of pro-apoptotic factors from mitochondria, such as cytochrome c. The released cytochrome c binds to the apoptotic protease-activating factor-1(Apaf-1), and subsequently turns on downstream executioner caspases such as caspase-3 [6, 7]. The anti-apoptotic and pro-apoptotic members of the different protein families play a critical role in STS induced apoptosis. Consistent with our previous studies, experimental data from the present study shows that PHB regulates both anti-apoptotic and pro-apoptotic members in STS induced apoptosis in undifferentiated GCs. STS treatment was found to up-regulate both mRNA and proteins of pro-apoptotic factors (Bax and Bak) greater than two fold with up-regulation of cytochrome-c release from mitochondria and increase caspases-3 activity in STS treated control or Ad-eGFP infected GCs. Interestingly, we did not observed any significant up-/down-regulation of other key regulatory factors such as IAP, Bid, Bad at the messenger levels under these experimental conditions.

The analysis of proteins regulating mitochondrial functions showed a strong correlation between the ratio of members of the Bcl-2 protein family, including Bax, Bak, Bclxl and Bcl-2, which determine the sensitivity of the GCs to STS-mediated apoptosis. In our undifferentiated GCs experimental model system, Bax/Bcl2, Bax/Bclxl, Bak/Bcl2 and Bak/Bclxl ratios of less than 70 % were characteristic for resistance to STS mediated apoptosis, whereas, Bax/Bcl2, Bax/Bclxl, Bak/Bcl2 and Bak/Bclxl ratios greater than 90 % were characteristic for the increased sensitivity of the GCs to STS. The apoptosis inhibiting effect of Bcl2 and Bclxl are counteracted by the pro-apoptotic proteins Bax and Bak. Imbalance of the Bax and Bak versus Bcl2 and Bclxl ratios tilts the scales toward cell death and sensitized cells to a wide variety of cell death stimuli, including all chemotherapeutic drugs, radiation, hypoxia, or growth factor withdrawal and enhance the resistance of cells to the cytotoxic effects [22–27]. Previous studies have demonstrated that ectopic Bcl2 over-expression in sensitive cell lines prevented the triggering of apoptotic stimuli, thereby supporting the role of the Bax/Bcl-2 rheostat as a key checkpoint [27, 28]. The broad resistance to cell death, occurring upon the intracellular balance of the Bax/Bcl2, Bax/Bclxl, Bak/Bcl2 and Bak/Bclxl ratios, have potential relevance for cell behavior including cell invasion, adhesion, or metastatic potential [29].

In agreement with these published observations, the current experimental data in this study have further demonstrated that PHB is also required for the phosphorylation of ERK which is involved in induction of the anti-apoptotic Bcl-2 factor. STS mediated phosphorylation of ERK1/2 is dependent on PHB protein expression and PHB is also required for MEK1 activity, suggesting that a possible novel regulatory loop affecting this pathway is mediated by PHB. Published studies have indicated that PHB plays an important role in the Ras-mediated activation of the Raf/MEK/ERK pathway [30, 31], which is a highly conserved signaling cascade that regulates a multitude of essential cellular functions such as proliferation and differentiation [31]. Interestingly, we also observed that STS induced phosphorylation of ERK suggesting an adaptive response to cellular stress, which is newly observed phenomenon that also occurs in undifferentiated GCs [32].

We detected a remarkable increment of the mitochondrial content of PHB in the STS treated undifferentiated GCs when compared to PHB knock down group. In the mitochondrial fraction, the concentration of PHB is much higher in STS treated GCs, suggesting that the increase of mitochondrial PHB is essential for stabilizing the mitochondrial integrity and maintaining mitochondrial membrane potential in theses rat ovarian undifferentiated GCs [8, 9, 33]. Mitochondria are dynamic structures that fuse and divide continuously to adjust the shape and distribution of the mitochondrial network which depends on cells state, stage and energy demands, and therefore, plays critical roles in cell physiology. The mitochondrial network is composed of highly interconnected tubules formed by balanced fusion and fission events [34]. Our immunofluorescence study revealed that a transition of mitochondrial morphology occurs from a reticular network to vesicular punctiform or fragmentation following knockdown of PHB with STS treatment. A decrease in the mitochondrial reticular network connectivity is an early apoptotic signal [35], that was also demonstrated in PHB-deficient MEFs and PHB- or PHB2-silenced cells [36, 37]. These collective observations suggest that PHB is an essential mitochondrial protein that is involved in maintaining mitochondrial integrity by promoting the fusion of mitochondrial membranes. The mechanism(s) involved in participation of PHB in the mitochondrial fusion/fission during the mitochondrial fragmentation leading to apoptosis may be explained by altered processing of OPA1 [38; our unpublished study]. OPA1 is a large dynamin-like GTPase that is found in the mitochondrial intermembrane space and regulates both mitochondrial fusion and cristae morphogenesis [35]. Thus, the reduced PHB content in mitochondria that occurs in PHB knock down in undifferentiated GCs may be one of the mechanisms to explain why changes in expression of PHB affects mitochondrial integrity and membrane potential in these cells [8, 9, 38, 39]. However, the detailed mechanism by which PHBs affect OPA1 processing still remains to be determined.

Thus, upon induction of apoptosis by STS, mitochondrial PHB through pPHB mediate activation of pERK expression cascade that results in enhancement of the Bcl/Bcl-xL pathway and inhibition of Bax-Bak, which directly inhibits the release of cytochrome c from the inter-mitochondrial space resulting in inhibition of the downstream activation of cleaved caspase 3. An alternative mechanism leading to inhibition of apoptosis after mitochondrial PHB over-expression may, in part, be due to the prevention of cytochrome c translocation into the cytosol thus protecting the mitochondria from undergoing functional incapacitation. The increased expression and translocation of PHB in the mitochondria in response to apoptotic signals and the delayed response to apoptotic stimulus that was observed in the current studies supports the role of PHB as an anti-apoptotic agent [8, 9]. Furthermore, these studies suggest that over-expression of PHB in GCs may inhibit the rate of oocyte depletion observed in females prior to menopause through the proposed pMEK-pERK-Bcl2/Bcl-xL signaling mechanisms. Thus, PHB may act either as a sensor for mitochondrial integrity and homeostasis and/or as a survival factor that paradoxically mediates GCs fate decisions during cellular proliferation and differentiation by delaying the activation of the apoptotic pathways, to support preantral and early antral follicular development in the ovary. These studies have demonstrated that the adenovirus system utilized in this study are a useful tool for facilitating future studies that are designed to decipher the molecular mechanisms involved in PHB mediated regulation of differentiation and proliferation in undifferentiated GCs.
